# 2,4-Dichloro­quinoline

**DOI:** 10.1107/S160053681001576X

**Published:** 2010-05-08

**Authors:** Roman Kimmel, Marek Nečas, Robert Vícha

**Affiliations:** a Department of Chemistry, Faculty of Technology, Tomas Bata University in Zlin, Nám. T.G. Masaryka 275, Zlín,762 72, Czech Republic; b Department of Chemistry, Faculty of Science, Masaryk University in Brno, Kamenice 5, Brno-Bohunice, 625 00, Czech Republic

## Abstract

The asymmetric unit of the title compound, C_9_H_5_Cl_2_N, consists of two crystallographically independent mol­ecules. In both mol­ecules the quinoline ring system is essentially planar [maximum deviations from the best plane of 0.0232 (13) 0.0089 (15) Å]. The angle between these planes is 22.40 (3)°. Conformers *A* and *B* are arranged face-to-face along the *c* axis, forming alternating pairs in the order *AABB*. The inter­planar distances *AA*, *AB* and *BB* are 3.3166 (11), 3.2771 (11) and 3.3935 (11) Å, respectively. The crystal packing is stabilized by weak C—H⋯Cl and C—H⋯N inter­actions.

## Related literature

For previous syntheses of title compound, see: Baeyer & Bloem (1882 ▶); Steinschifter & Stadlbauer (1994 ▶). For the use of the title compound in organic synthesis, see: Buchmann & Hamilton (1942 ▶).
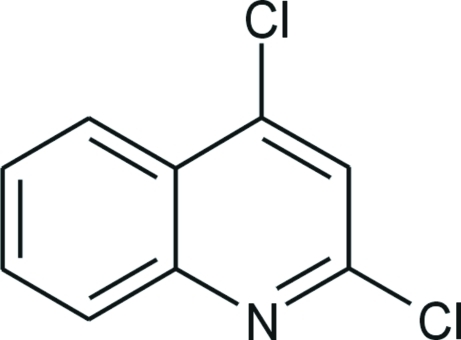


## Experimental

### 

#### Crystal data

C_9_H_5_Cl_2_N
*M* *_r_* = 198.04
Monoclinic, 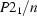

*a* = 10.3689 (3) Å
*b* = 11.9215 (3) Å
*c* = 13.6380 (5) Å
β = 98.937 (3)°
*V* = 1665.37 (9) Å^3^
*Z* = 8
Mo *K*α radiation
μ = 0.71 mm^−1^
*T* = 120 K
0.40 × 0.40 × 0.30 mm

#### Data collection

Kuma KM-4-CCD diffractometer
Absorption correction: multi-scan (*CrysAlis RED*; Oxford Diffraction, 2006 ▶) *T*_min_ = 0.60, *T*_max_ = 0.81
13224 measured reflections
2927 independent reflections
2504 reflections with *I* > 2σ(*I*)
*R*_int_ = 0.012

#### Refinement

*R*[*F* ^2^ > 2σ(*F* ^2^)] = 0.021
*wR*(*F* ^2^) = 0.066
*S* = 1.08
2927 reflections
217 parameters
H-atom parameters constrained
Δρ_max_ = 0.25 e Å^−3^
Δρ_min_ = −0.22 e Å^−3^

### 

Data collection: *CrysAlis CCD* (Oxford Diffraction, 2006 ▶); cell refinement: *CrysAlis CCD*; data reduction: *CrysAlis RED* (Oxford Diffraction, 2006 ▶); program(s) used to solve structure: *SHELXS97* (Sheldrick, 2008 ▶); program(s) used to refine structure: *SHELXL97* (Sheldrick, 2008 ▶); molecular graphics: *ORTEP-3* (Farrugia, 1997 ▶) and *Mercury* (Macrae *et al.*, 2008 ▶); software used to prepare material for publication: *SHELXL97*.

## Supplementary Material

Crystal structure: contains datablocks global, I. DOI: 10.1107/S160053681001576X/bg2343sup1.cif

Structure factors: contains datablocks I. DOI: 10.1107/S160053681001576X/bg2343Isup2.hkl

Additional supplementary materials:  crystallographic information; 3D view; checkCIF report

## Figures and Tables

**Table 1:** Hydrogen-bond geometry (Å, °)

*D*—H⋯*A*	*D*—H	H⋯*A*	*D*⋯*A*	*D*—H⋯*A*
C2—H2*A*⋯Cl4	0.95	2.88	3.7197 (14)	148
C17—H17*A*⋯N1^i^	0.95	2.60	3.5111 (19)	162
C18—H18*A*⋯Cl1^i^	0.95	2.95	3.7290 (15)	141

Symmetry code: (i) 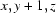.

**Fig. 1.**
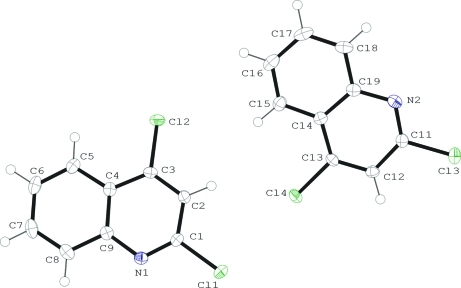
Ellipsoid plot of the asymmetric unit with atoms represented as 50% probability ellipsoids.

**Fig. 2.**
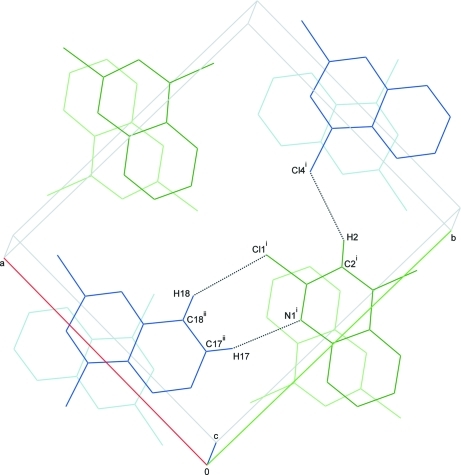
Eight molecules lying around an inversion centre and viewed along the c axis are coloured by symmetry equivalence. The H-bond cross-linkage framework is drawn in the front layer by dotted lines.Hydrogen atoms are omitted except for those participating in H-bonds. Symmetry codes: (i) -x+0.5, y+0.5,-z+0.5; (ii) -x+0.5, y-0.5, -z+0.5.

## supplementary crystallographic information

### Comment

Although the 2,4-dichloroquinoline is well known for more than hundred years (Baeyer & Bloem, 1882) and has been widely used in quinoline chemistry (Buchmann & Hamilton, 1942; Steinschifter & Stadlbauer, 1994), no structure data has been published so far.

The title compound (Fig. 1) crystallises with two crystallographical independent molecules in asymmetric unit. Conformers A and B differ very little in geometrical parameters. Both of them consist of essentially planar quinoline ring with maximum deviations from the best planes being 0.0232 (13) Å for atom C2 (conformer A) and 0.0089 (15) Å for atom C17 (conformer B). The angle between these quinoline best planes is 22.40 (3)°. Chlorine atoms lay almost in the ring best planes with the deviations 0.0035 (4) Å for atom Cl1 and -0.0011 (4) for atom Cl2 (conformer A) and -0.0081 (4) Å for atom Cl3 and 0.0121 (4) Å for atom Cl4 (conformer B). Pairs of conformers are stacked along the *c* axes in AABB arrangement stabilised via offset π–π interactions. The distances between AA, AB and BB planes calculated as a distance of nitrogen atom from adjacent ring plane are 3.3166 (11), 3.2771 (11) and 3.3935 (11) Å, respectively. Molecular packing is stabilised by C—H···Cl and C—H···N weak interactions (Fig. 2, Table 1).

### Experimental

4-Hydroxyquinolin-2-one (322 mg, 2 mmol) and POCl_3_ (2 ml) were treated for 15 min. at 100°C. Reaction mixture was poured onto finely crushed ice to decompose an excess of POCl_3_. Basicity was adjusted to pH =8 using Na_2_CO_3_ and resulting precipitate was filtered off. The solid on the filter was washed with water and dried at room temperature to yield 292 mg (74%) of title compound.The single crystal used for data collection was obtained by crystallisation from diethyl ether at room temperature.

### Crystal data

**Table:**

C_9_H_5_Cl_2_N	*F*(000) = 800
*M**_r_* = 198.04	*D*_x_ = 1.580 Mg m^−^^3^
Monoclinic, *P*2_1_/*n*	Melting point: 335(1) K
Hall symbol: -P 2yn	Mo *K*α radiation, λ = 0.71073 Å
*a* = 10.3689 (3) Å	Cell parameters from 14773 reflections
*b* = 11.9215 (3) Å	θ = 2.9–27.1°
*c* = 13.6380 (5) Å	µ = 0.71 mm^−^^1^
β = 98.937 (3)°	*T* = 120 K
*V* = 1665.37 (9) Å^3^	Block, yellow
*Z* = 8	0.40 × 0.40 × 0.30 mm

### Data collection

**Table:**

Kuma KM-4-CCD diffractometer	2927 independent reflections
Radiation source: fine-focus sealed tube	2504 reflections with *I* > 2σ(*I*)
graphite	*R*_int_ = 0.012
ω scan	θ_max_ = 25.0°, θ_min_ = 2.9°
Absorption correction: multi-scan (*CrysAlis RED*; Oxford Diffraction, 2006)	*h* = −12→12
*T*_min_ = 0.60, *T*_max_ = 0.81	*k* = −14→14
13224 measured reflections	*l* = −16→13

### Refinement

**Table:**

Refinement on *F*^2^	Primary atom site location: structure-invariant direct methods
Least-squares matrix: full	Secondary atom site location: difference Fourier map
*R*[*F*^2^ > 2σ(*F*^2^)] = 0.021	Hydrogen site location: inferred from neighbouring sites
*wR*(*F*^2^) = 0.066	H-atom parameters constrained
*S* = 1.08	*w* = 1/[σ^2^(*F*_o_^2^) + (0.041*P*)^2^ + 0.2572*P*] where *P* = (*F*_o_^2^ + 2*F*_c_^2^)/3
2927 reflections	(Δ/σ)_max_ = 0.005
217 parameters	Δρ_max_ = 0.25 e Å^−^^3^
0 restraints	Δρ_min_ = −0.22 e Å^−^^3^

### Special details

**Table:**

Geometry. All esds (except the esd in the dihedral angle between two l.s. planes) are estimated using the full covariance matrix. The cell esds are taken into account individually in the estimation of esds in distances, angles and torsion angles; correlations between esds in cell parameters are only used when they are defined by crystal symmetry. An approximate (isotropic) treatment of cell esds is used for estimating esds involving l.s. planes.
Refinement. Refinement of *F*^2^ against ALL reflections. The weighted *R*-factor wR and goodness of fit *S* are based on *F*^2^, conventional *R*-factors *R* are based on *F*, with *F* set to zero for negative *F*^2^. The threshold expression of *F*^2^ > σ(*F*^2^) is used only for calculating *R*-factors(gt) etc. and is not relevant to the choice of reflections for refinement. *R*-factors based on *F*^2^ are statistically about twice as large as those based on *F*, and *R*- factors based on ALL data will be even larger.

### Fractional atomic coordinates and isotropic or equivalent isotropic displacement parameters (Å^2^)

**Table:**

	*x*	*y*	*z*	*U*_iso_*/*U*_eq_	
Cl1	0.13384 (3)	0.03305 (3)	0.08803 (3)	0.02540 (11)	
Cl2	0.52857 (3)	0.32412 (3)	0.17438 (3)	0.02529 (11)	
Cl3	−0.36457 (3)	0.59016 (3)	0.06753 (3)	0.02745 (11)	
Cl4	0.02870 (3)	0.30160 (3)	0.16841 (3)	0.02323 (11)	
C1	0.30194 (13)	0.05798 (11)	0.10998 (10)	0.0179 (3)	
C2	0.34206 (13)	0.16982 (11)	0.12943 (10)	0.0184 (3)	
H2A	0.2808	0.2290	0.1297	0.022*	
C3	0.47285 (13)	0.18864 (11)	0.14782 (10)	0.0170 (3)	
C4	0.56380 (13)	0.09974 (11)	0.14534 (9)	0.0174 (3)	
C5	0.70140 (13)	0.11242 (12)	0.16231 (10)	0.0218 (3)	
H5A	0.7389	0.1842	0.1779	0.026*	
C6	0.78035 (14)	0.02124 (13)	0.15621 (11)	0.0262 (3)	
H6A	0.8725	0.0303	0.1677	0.031*	
C7	0.72627 (14)	−0.08561 (13)	0.13307 (10)	0.0252 (3)	
H7A	0.7821	−0.1479	0.1285	0.030*	
C8	0.59391 (14)	−0.10019 (12)	0.11718 (10)	0.0217 (3)	
H8A	0.5585	−0.1728	0.1021	0.026*	
C9	0.50939 (13)	−0.00843 (11)	0.12290 (9)	0.0171 (3)	
N1	0.37727 (11)	−0.02819 (9)	0.10649 (8)	0.0178 (3)	
C11	−0.19672 (13)	0.56562 (11)	0.09168 (10)	0.0185 (3)	
C12	−0.15678 (13)	0.45494 (11)	0.11711 (10)	0.0179 (3)	
H12A	−0.2182	0.3964	0.1204	0.022*	
C13	−0.02595 (13)	0.43615 (11)	0.13660 (9)	0.0165 (3)	
C14	0.06551 (13)	0.52382 (11)	0.13046 (9)	0.0175 (3)	
C15	0.20256 (13)	0.51151 (12)	0.14938 (10)	0.0217 (3)	
H15A	0.2400	0.4406	0.1684	0.026*	
C16	0.28175 (14)	0.60210 (13)	0.14029 (11)	0.0274 (3)	
H16A	0.3739	0.5931	0.1530	0.033*	
C17	0.22853 (15)	0.70773 (13)	0.11250 (11)	0.0281 (4)	
H17A	0.2847	0.7694	0.1063	0.034*	
C18	0.09590 (15)	0.72227 (12)	0.09421 (10)	0.0248 (3)	
H18A	0.0607	0.7941	0.0756	0.030*	
C19	0.01108 (13)	0.63129 (11)	0.10277 (10)	0.0183 (3)	
N2	−0.12097 (11)	0.65102 (9)	0.08387 (8)	0.0200 (3)	

### Atomic displacement parameters (Å^2^)

**Table:**

	*U*^11^	*U*^22^	*U*^33^	*U*^12^	*U*^13^	*U*^23^
Cl1	0.01550 (19)	0.0246 (2)	0.0355 (2)	−0.00423 (13)	0.00217 (15)	−0.00300 (15)
Cl2	0.0226 (2)	0.01604 (18)	0.0354 (2)	−0.00448 (13)	−0.00113 (16)	−0.00104 (14)
Cl3	0.0184 (2)	0.0270 (2)	0.0358 (2)	0.00475 (14)	0.00067 (15)	0.00256 (16)
Cl4	0.0236 (2)	0.01571 (18)	0.0307 (2)	0.00392 (13)	0.00519 (15)	0.00299 (13)
C1	0.0150 (7)	0.0223 (7)	0.0163 (7)	−0.0019 (5)	0.0016 (6)	0.0001 (5)
C2	0.0180 (7)	0.0169 (7)	0.0202 (7)	0.0016 (5)	0.0027 (6)	0.0009 (5)
C3	0.0210 (7)	0.0151 (7)	0.0145 (7)	−0.0023 (5)	0.0011 (6)	0.0006 (5)
C4	0.0188 (7)	0.0208 (7)	0.0122 (7)	0.0017 (5)	0.0016 (5)	0.0024 (5)
C5	0.0175 (7)	0.0274 (8)	0.0203 (7)	−0.0019 (6)	0.0021 (6)	0.0027 (6)
C6	0.0165 (7)	0.0394 (9)	0.0225 (8)	0.0053 (6)	0.0020 (6)	0.0050 (7)
C7	0.0258 (8)	0.0320 (8)	0.0179 (8)	0.0129 (6)	0.0038 (6)	0.0032 (6)
C8	0.0294 (8)	0.0197 (7)	0.0162 (7)	0.0053 (6)	0.0043 (6)	0.0007 (6)
C9	0.0201 (7)	0.0202 (7)	0.0108 (7)	0.0016 (6)	0.0019 (5)	0.0022 (5)
N1	0.0198 (6)	0.0172 (6)	0.0161 (6)	−0.0011 (5)	0.0018 (5)	0.0001 (5)
C11	0.0183 (7)	0.0204 (7)	0.0167 (7)	0.0016 (5)	0.0023 (6)	−0.0016 (5)
C12	0.0195 (7)	0.0175 (7)	0.0174 (7)	−0.0023 (5)	0.0047 (6)	−0.0007 (5)
C13	0.0212 (7)	0.0149 (7)	0.0134 (7)	0.0020 (5)	0.0033 (6)	−0.0004 (5)
C14	0.0205 (7)	0.0195 (7)	0.0131 (7)	−0.0012 (6)	0.0041 (6)	−0.0033 (5)
C15	0.0192 (7)	0.0259 (8)	0.0201 (7)	0.0003 (6)	0.0037 (6)	−0.0035 (6)
C16	0.0198 (8)	0.0365 (9)	0.0263 (8)	−0.0075 (6)	0.0048 (6)	−0.0073 (7)
C17	0.0292 (9)	0.0296 (8)	0.0261 (8)	−0.0146 (7)	0.0060 (7)	−0.0049 (7)
C18	0.0336 (9)	0.0177 (7)	0.0234 (8)	−0.0061 (6)	0.0060 (6)	−0.0029 (6)
C19	0.0228 (7)	0.0186 (7)	0.0138 (7)	−0.0017 (6)	0.0039 (6)	−0.0035 (5)
N2	0.0239 (7)	0.0165 (6)	0.0198 (6)	0.0008 (5)	0.0040 (5)	−0.0006 (5)

### Geometric parameters (Å, °)

**Table:**

Cl1—C1	1.7475 (13)	C8—H8A	0.9500
Cl2—C3	1.7345 (13)	C9—N1	1.3738 (17)
Cl3—C11	1.7450 (14)	C11—N2	1.3003 (18)
Cl4—C13	1.7338 (13)	C11—C12	1.4100 (18)
C1—N1	1.2959 (17)	C12—C13	1.3598 (19)
C1—C2	1.4096 (18)	C12—H12A	0.9500
C2—C3	1.3589 (19)	C13—C14	1.4225 (19)
C2—H2A	0.9500	C14—C15	1.4120 (19)
C3—C4	1.4225 (18)	C14—C19	1.4270 (19)
C4—C5	1.4175 (19)	C15—C16	1.374 (2)
C4—C9	1.4217 (19)	C15—H15A	0.9500
C5—C6	1.371 (2)	C16—C17	1.403 (2)
C5—H5A	0.9500	C16—H16A	0.9500
C6—C7	1.408 (2)	C17—C18	1.370 (2)
C6—H6A	0.9500	C17—H17A	0.9500
C7—C8	1.367 (2)	C18—C19	1.4126 (19)
C7—H7A	0.9500	C18—H18A	0.9500
C8—C9	1.4114 (19)	C19—N2	1.3736 (18)
			
N1—C1—C2	126.50 (12)	N2—C11—C12	126.50 (13)
N1—C1—Cl1	116.73 (10)	N2—C11—Cl3	116.78 (10)
C2—C1—Cl1	116.77 (10)	C12—C11—Cl3	116.72 (10)
C3—C2—C1	116.60 (12)	C13—C12—C11	116.62 (12)
C3—C2—H2A	121.7	C13—C12—H12A	121.7
C1—C2—H2A	121.7	C11—C12—H12A	121.7
C2—C3—C4	121.26 (12)	C12—C13—C14	121.43 (12)
C2—C3—Cl2	118.87 (10)	C12—C13—Cl4	118.60 (10)
C4—C3—Cl2	119.88 (10)	C14—C13—Cl4	119.97 (10)
C5—C4—C3	124.81 (12)	C15—C14—C13	125.04 (13)
C5—C4—C9	119.18 (12)	C15—C14—C19	119.15 (12)
C3—C4—C9	116.01 (12)	C13—C14—C19	115.81 (12)
C6—C5—C4	120.06 (13)	C16—C15—C14	120.03 (14)
C6—C5—H5A	120.0	C16—C15—H15A	120.0
C4—C5—H5A	120.0	C14—C15—H15A	120.0
C5—C6—C7	120.67 (13)	C15—C16—C17	120.95 (14)
C5—C6—H6A	119.7	C15—C16—H16A	119.5
C7—C6—H6A	119.7	C17—C16—H16A	119.5
C8—C7—C6	120.38 (13)	C18—C17—C16	120.28 (13)
C8—C7—H7A	119.8	C18—C17—H17A	119.9
C6—C7—H7A	119.8	C16—C17—H17A	119.9
C7—C8—C9	120.63 (13)	C17—C18—C19	120.54 (14)
C7—C8—H8A	119.7	C17—C18—H18A	119.7
C9—C8—H8A	119.7	C19—C18—H18A	119.7
N1—C9—C8	118.04 (12)	N2—C19—C18	117.92 (12)
N1—C9—C4	122.89 (12)	N2—C19—C14	123.03 (12)
C8—C9—C4	119.07 (12)	C18—C19—C14	119.04 (12)
C1—N1—C9	116.71 (11)	C11—N2—C19	116.59 (11)

### Hydrogen-bond geometry (Å, °)

**Table:**

*D*—H···*A*	*D*—H	H···*A*	*D*···*A*	*D*—H···*A*
C2—H2A···Cl4	0.95	2.88	3.7197 (14)	148
C17—H17A···N1^i^	0.95	2.60	3.5111 (19)	162
C18—H18A···Cl1^i^	0.95	2.95	3.7290 (15)	141

Symmetry codes: (i) *x*, *y*+1, *z*.
